# MicroRNA profiles reveal female allotetraploid hybrid fertility

**DOI:** 10.1186/s12863-015-0276-y

**Published:** 2015-10-14

**Authors:** Rong Zhou, Yanhong Wu, Min Tao, Chun Zhang, Shaojun Liu

**Affiliations:** Key Laboratory of Protein Chemistry and Developmental Biology of the State Education Ministry of China, College of Life Sciences, Hunan Normal University, Changsha, Hunan 410081 People’s Republic of China

**Keywords:** MicroRNA, Fertility, Polyploidy, High throughout sequencing

## Abstract

**Background:**

The bisexual fertile tetraploid fish is important in biological evolution. Tetraploid fish fertility is the key factor for stable inheritance. Therefore, elucidating tetraploid fish fertility at the molecular level is essential. MicroRNAs regulate gene expression and are involved in many aspects of gonad development.

**Methods:**

Total RNA was isolated using TRIzol, followed by constructing small RNA libraries. And then, the qualified libraries were sequenced with the HiSeq 2500 SE50 system. The obtained clean reads were analyzed to identify conserved and novel miRNAs, and evaluate the expression, and also predict the target genes. The differential expressions of miRNAs were confirmed by RT-PCR.

**Results:**

In this study, allotetraploid hybrid fish (*4n*AT) and diploid red crucian carp (RCC) ovaries were used to compare miRNA profiles. The results indicated that most of the highly expressed miRNAs were closely correlated with ovary maturation, and displayed no significant differences in expression. Moreover, 34 up-regulated and nine down-regulated miRNAs were found in *4n*AT. The differentially expressed miRNAs were primarily involved in metabolism, defense mechanisms, and cytoskeleton production.

**Conclusions:**

This is the first study to provide new epigenetic evidences for tetraploid fish fertility and phenotypic changes as a result of increased ploidy.

**Electronic supplementary material:**

The online version of this article (doi:10.1186/s12863-015-0276-y) contains supplementary material, which is available to authorized users.

## Background

Polyploidization is one of important features of species evolution [1, 2]. Duplication of the whole genome was proved to be occurred during long-term evolution of many eukaryotes [3]. The polyploidy phenomenon, arose when a rare meiotic or mitotic event resulted in the formation of gametes with more than one set of chromosomes, is common in plants but rare in animals, especially vertebrates [4, 5]. Thus, polyploid organisms contain at least three or more complete chromosome sets, with four being the most common (tetraploidy) [5]. Polyploidy is divided into autopolyploidy and allopolyploidy. Allopolyploidy involves distant hybridization and duplication of divergent parental genomes, this induces more rapid genome evolution than autopolyploidy [6]. For survival, the allopolyploid individual, with more than two differential genomes in the same nucleus, must experience challenges and balance the extra biochemical diversity and gene expression of multiple genomes [7]. For inheritance, the allotetraploid individuals must be bisexual fertile to maintain the genetic characteristics from one generation to the next. Therefore, it is essential to prove the fertility of polyploid individuals at the morphological and molecular levels before studying their special roles in biological evolution.

The allotetraploid hybrid (*4n*AT) was derived from the distant hybridization of female red crucian carp (RCC) and male common carp (CC), which is the first bisexual fertile allotetraploid fish, even vertebrate reported so far [8]. In the F_2_ hybrid lineage, the hybrid offspring could produce unreduced diploid gametes [9]. Thus, self-crossing of F_2_ resulted in the appearance of allotetraploid hybrids in F_3_, which is then stably inherited through to F_24_ [10]. The cytogenetic studies revealed that *4n*AT possessed 200 chromosomes, which form 100 bivalents during meiosis I to produce stable diploid gametes [11, 12]. Observations of gonad tissue sections have revealed that *4n*AT was matured at 1 year and experienced a similar gonad developmental process as diploid RCC [10, 11]. Molecular evidences have shown that regulation of reproductive endocrine in *4n*AT is normal [13–15]. All these studies have shown that *4n*AT is bisexual fertile. However, the epigenetic regulation of reproductive characteristics (e.g., fertility and gamete ploidy) between allotetraploid and diploid fishes has not been studied to date.

MicroRNA is a kind of highly conserved endogenous noncoding small RNA approximately 22 nt in length, first discovered in the early 1990s by Lee and Wightman in *C. elegans* in which it regulates heterochronic gene *lin-14* expression to mediate temporal pattern formation [16, 17]. Mature miRNAs are formed through three sequential steps: (1) the longer nascent transcripts (termed pri-miRNAs) are transcribed by RNA polymerase II in the nucleus [18]; (2) the ~70 nt long precursors of miRNAs (termed pre-miRNAs) are generated in the nucleus by Drosha and then transported to the cytoplasm [19]; (3) the ~22 nt long mature miRNAs are formed via processing by Dicer in the cytoplasm [20]. MiRNA, as an important epigenetic modification, regulates gene expression by recognizing and binding 3’-untranslated regions of target mRNAs to either block gene translation or induce mRNA cleavage [21]. Target deletion of Dicer 1 in mouse ovaries provided the first empirical evidence that miRNAs are critical for the normal development of the female reproductive system and fertility [22, 23]. Moreover, a series of gonad-specific expressed miRNAs have been identified through the comparison of gonad and other tissues, or different gonad developmental stages both in mammals and teleost fishes [24–26].

In this study, we compared the miRNA profiles of diploid RCC and *4n*AT ovaries, and found several differentially expressed miRNAs, including 34 up-regulated and nine down-regulated in *4n*AT. Target gene prediction and functional annotation analysis revealed that genes targeted by differentially expressed miRNAs were primarily involved in the metabolic, cytoskeleton, and defense systems. However, miRNAs related to ovary maturation were abundant and exhibited similar expression levels in both groups of samples. This study provides epigenetic evidences for female *4n*AT fertility and phenotypic changes resulting from increased ploidy.

## Results

### General description of miRNA libraries

In this study, we chose diploid RCC and *4n*AT ovaries to analyze gene expression and regulation coupled with species evolution driven by polyploidy. In each group, three individuals were used as biological replicates. Thus, six miRNA libraries were constructed in total, followed by high throughput sequencing. General bioinformatic analysis revealed that on average 15.521 M and 15.28 M raw reads were obtained in the two groups, respectively. After filtering by sequence length (18–30 nt) and removing the low quality reads including those containing “N”, 11.819 M and 12.414 M clean reads were obtained for the subsequent analysis. The detailed read numbers of each sample were shown in Additional file 1: Table S1.

The zebrafish genome was selected as the reference genome. Blasted against the GenBank and Rfam databases, ~12 % clean reads in diploid RCC and 14 % in *4n*AT were mapped to the genome with the standard of permitting no more than one base pair mismatch. Percentages of other types of RNAs (including rRNA, snRNA, snoRNA, and tRNA) and unmapped sequences were shown in Additional file 1: Table S2.

**Fig. 1:**
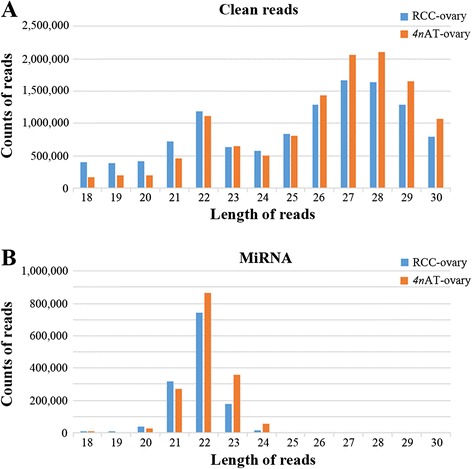
Length distribution of clean reads (**a**) and miRNAs (**b**)

**Fig. 2:**
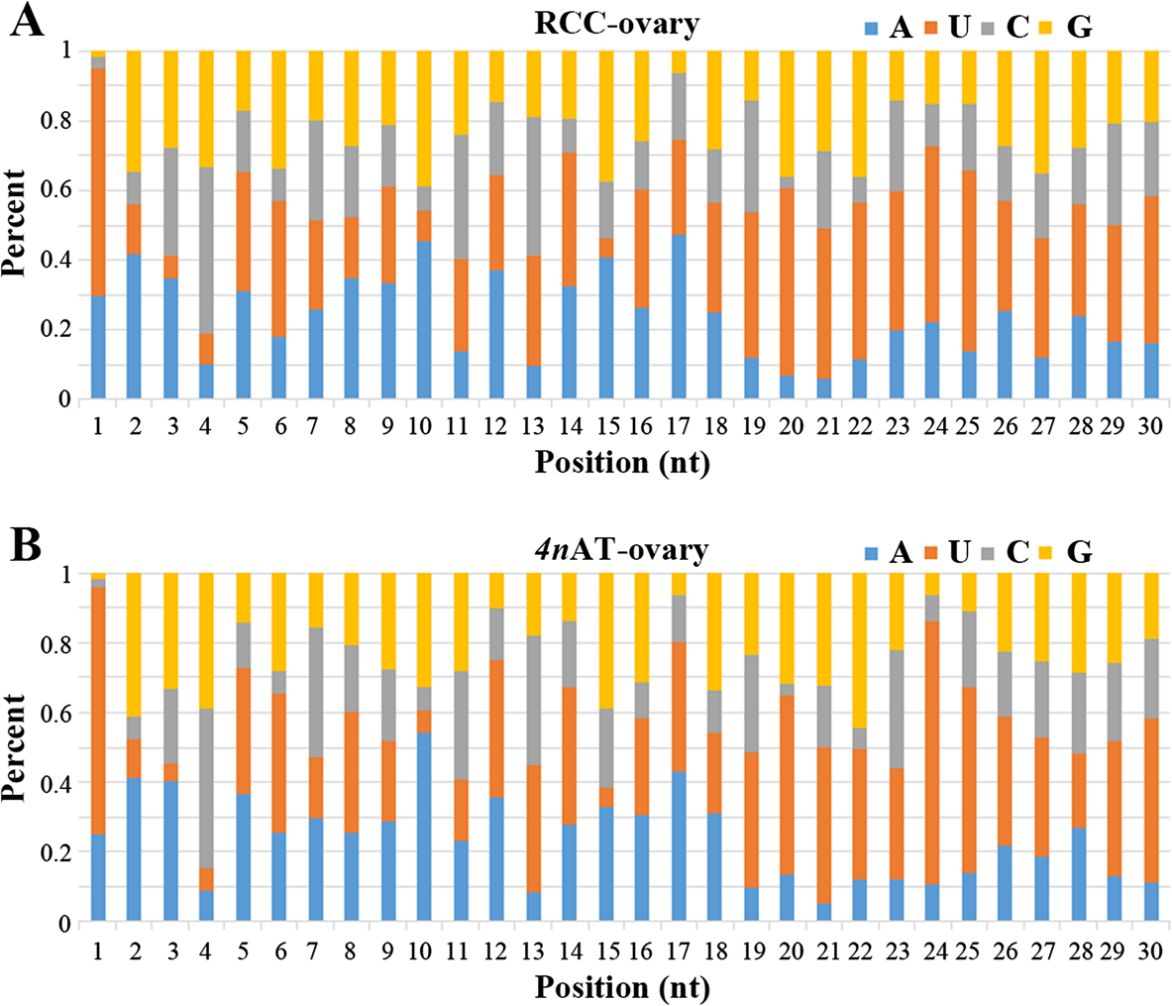
Base bias of small RNA at each position in diploid RCC (**a**) and *4n*AT (**b**)

The length distribution of clean reads mainly concentrated in two regions, 21–23 nt and 26–29 nt (Fig. 1a). However, the 21–23 nt reads were most likely to be the miRNAs (Fig. 1b). Statistical analysis of the base sequences indicated that the first base is biased to “U”, the tenth base biased to “A”, and the probability of “U” at the second to forth bases was very low (Fig. 2).

### Identification of distinct miRNAs

To identify the conserved and novel miRNAs, we used the miRDeep2 software for mapping the small RNA sequences to the reference genome. The results revealed that there were 441 mature miRNAs in diploid RCC, including 414 conserved and 30 novel miRNAs; 418 mature miRNAs were identified in *4n*AT, including 377 conserved and 41 novel miRNAs.

The composition and expression patterns of miRNAs are closely correlated with the biological characteristics of tissues. High throughput sequencing evaluated the frequency of different miRNAs in different samples. In both diploid RCC and *4n*AT ovaries, miR-143-3p exhibited the highest expression with approximately 145,643 reads on average; followed by miR-22-3p, miR-51-5p, miR-202-5p and let-7-5p with over 70,000 reads (Additional file 1: Table S3). In contrast, some miRNAs expressed less than five reads on average, such as miR-683, miR-4257, miR-4030-3p, miR-211-3p, miR-3243, and miR-3920 (Additional file 1: Table S3). This result revealed that the expression levels of different miRNAs were varied greatly.

### Differential expressed miRNAs in 4nAT as compared to diploid RCC

To investigate the differential expression of miRNAs in the ovaries of different ploidy fishes, correlations among the biological replicates in each group were first analyzed. Triplicate diploid RCC were closely correlated with R^2^ > 0.92. In the three *4n*AT replicates, samples four and five were closely correlated with R^2^ = 0.95, while sample six was not closely correlated with the other two R^2^ = 0.78 and 0.69, respectively (Additional file 1: Table S4, Additional file 2: Figure S1). This might be the result of individual differences. Thus, sample six of the *4n*AT group was excluded from the subsequent analysis.

**Fig. 3:**
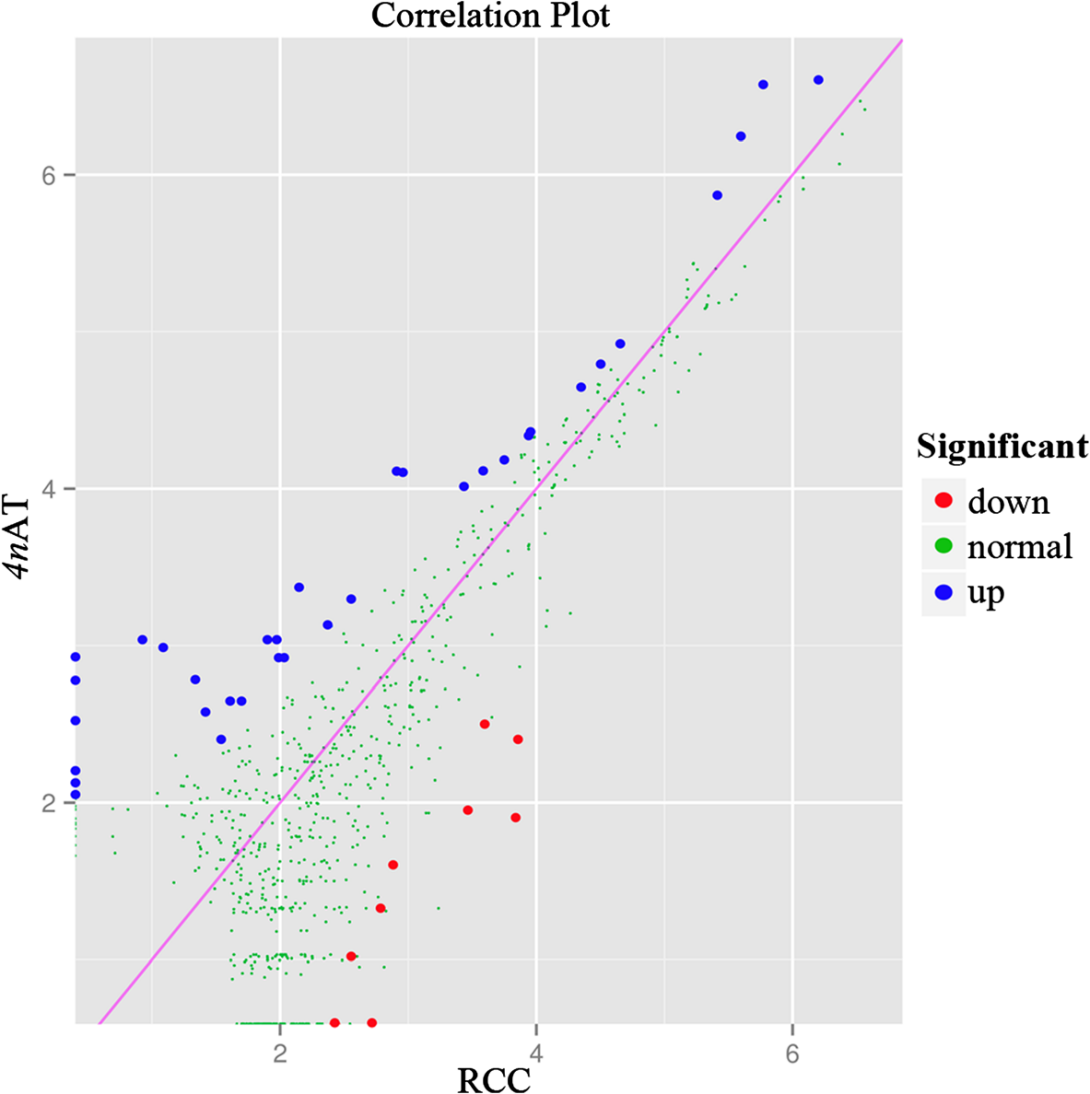
Comparison of expression of miRNAs between diploid RCC and *4n*AT

The main objective of this study is to illustrate the differential expression of miRNAs in the ovaries of different ploidy fishes. The relative expression level of each miRNA could be calculated according to the deep sequencing results. The differential expressions of miRNAs were screened with the standard of false discovery rate (FDR) <0.01 and fold change (FC) >2. We found that 9 conserved miRNAs were down-regulated, whereas 30 conserved and 4 non-conserved miRNAs were up-regulated in *4n*AT (Fig. 3, Additional file 1: Table S5). Among the down-regulated miRNAs, miR-6843-3p exhibited the greatest fold change, followed by miR-1738 and miR-3526. Whereas among the up-regulated miRNAs, miR-2285q exhibited the greatest fold change, followed by miR-503-5p, miR-3183, miR-301a-5p, miR-2492-3p, miR-63 k-3p, miR-1421 m-5p, and miR-3086-5p (Additional file 1: Table S5).

**Fig. 4:**
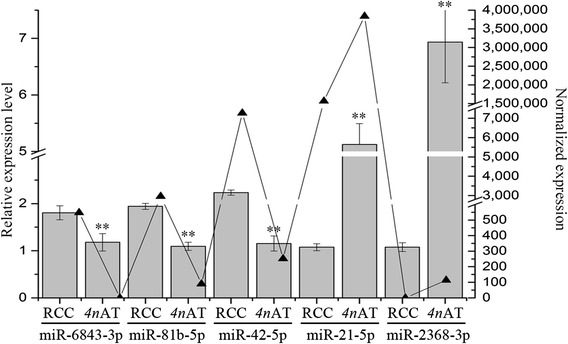
The relationship between relative expression levels of miRNA validated by RT-PCR and normalized expression derived from sequencing. Column charts depicted the expression levels of miRNAs measured by RT-PCR. The triangles represented the expression after normalization from sequencing

To validate the relative differential expressions based on high throughput sequencing, five differentially expressed miRNAs (miR-6843-3p, miR-81b-p, miR-42-5p, miR-21-5p, and miR-2368-3p) were analyzed for relative expression levels by quantitative real-time PCR in the ovaries of different ploidy fishes (Fig. 4). The real-time PCR results were consistent with those of the high throughput sequencing.

### Target gene prediction and function annotation

To illustrate the biological processes and physiological functions in which the differentially expressed miRNAs involved, the target gene sequences were predicted by the miRanda database. Thirty-nine unigenes targeted by up-regulated miRNAs were identified, but no gene information corresponding to down-regulated miRNAs were found (Additional file 1: Table S6). The function annotation and module classification of target genes were carried out by BLAST searches against the NR, Swiss-Prot, GO, COG, and KEGG databases.

**Fig. 5:**
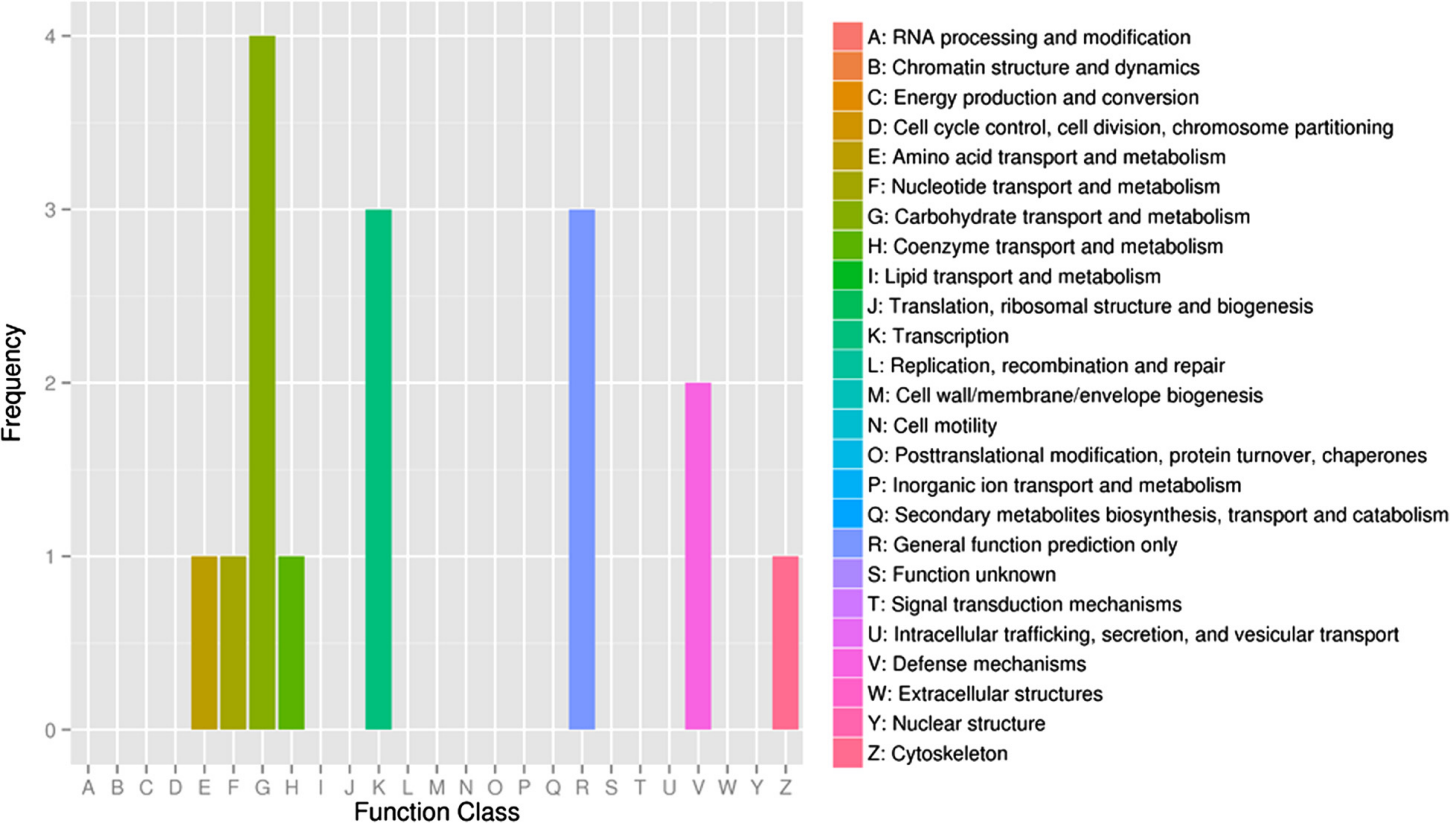
Function annotation of genes targeted by differential expressed miRNAs with COG database

Annotation with the COG database revealed that the differentially expressed target genes were mainly assigned to macromolecular transport and metabolism (e.g., amino acids, nucleotides, carbohydrates, and coenzymes), transcription, defense mechanisms, cytoskeleton production, and other general functions (Fig. 5).

**Fig. 6:**
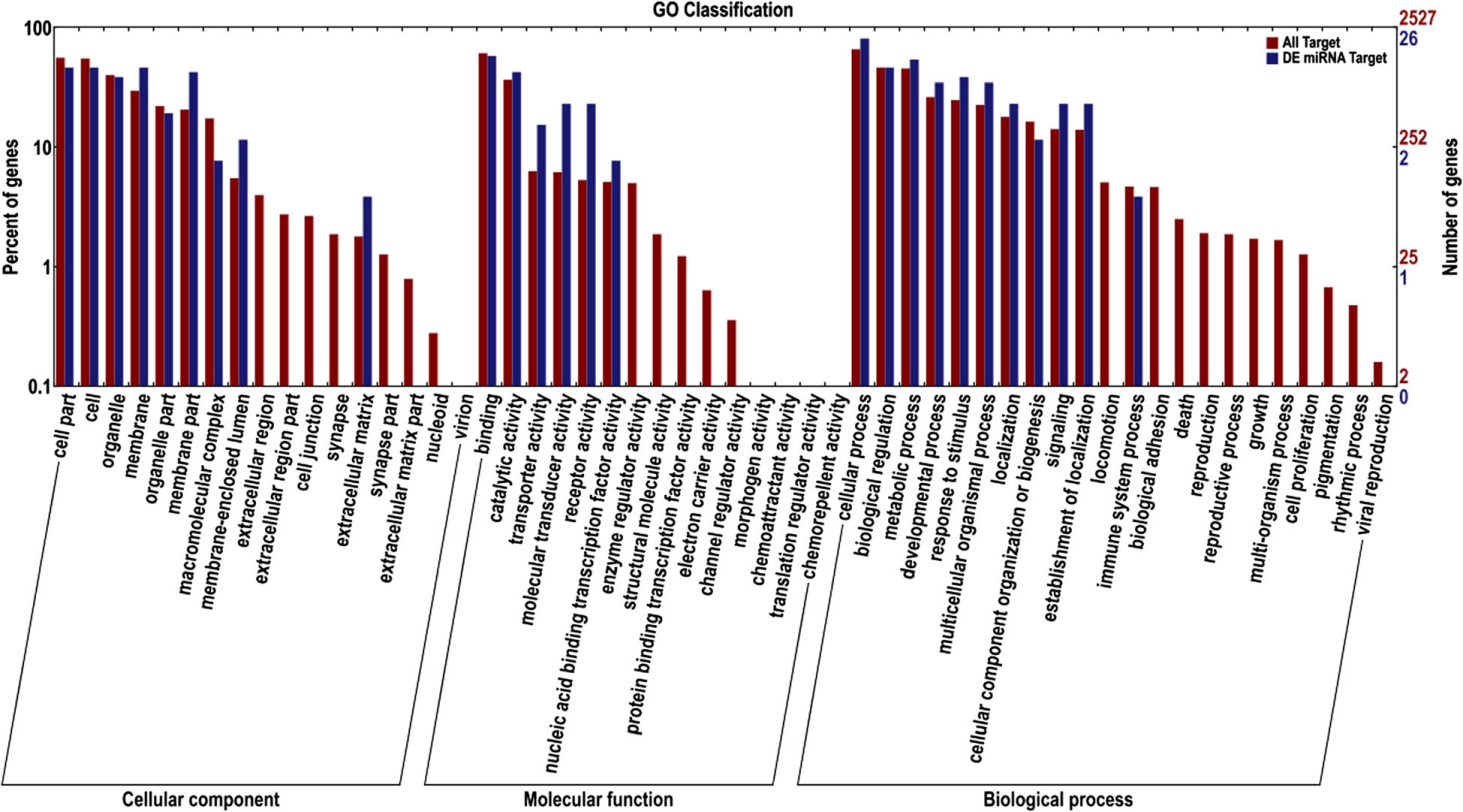
GO classification of functions of all predicted target genes and differential expressed miRNAs targeted genes

GO analysis indicated that the differentially expressed target genes were distributed in the following three classifications (Fig. 6). As for the cellular component, there were more differentially expressed target genes in the intracellular parts (e.g., cell, organelles, membranes, macromolecular complex, and membrane-enclosed lumen) and extracellular matrix. Regarding molecular function, there were more differentially expressed target genes involved in in binding, catalytic activity, transporter activity, molecular transducer activity, receptor activity, and nucleic acid binding transcription factor activity. As for the biological and cellular processes, biological regulation, metabolic processes, developmental processes, response to stimulus, multicellular organismal processes, localization, cellular component organization or biogenesis, signaling, establishment of localization, and immune system processes had higher numbers of differentially expressed target genes.

KEGG pathway analysis revealed that the differentially expressed target genes mainly focused on 11 signaling pathways, such as metabolic pathways (glycine, serine, threonine, galactose, starch, sucrose, amino sugar, nucleotide sugar, porphyrin, and chlorophyll metabolism), ABC transporters, one carbon pool by folate, pentose and glucoronate interconversions, lysosome, neuroactive ligand-receptor interaction, and the calcium signaling pathway (Additional file 1: Table S7).

## Discussion

Polyploidization is one of important features of species evolution. Distant hybridization is a useful strategy for transferring the genome of one species to another, thus resulting in hybrid vigor (phenotype) and polyploidy formation (genotype) in the offspring [10]. Through combining distant hybridization, genetic breeding, and self-crossing, the allotetraploid hybrid line has been established from F_3_ to F_24_, these artificially cultured polyploid fish with stable inheritance are the first bisexual fertility allotetraploid fish, even vertebrate [8]. Molecular genetic marker analysis, including AFLP and ISSR, between *4n*AT and their parents revealed that the genetic characteristics of the *4n*AT population is stable and represent a bias to the maternal progenitor [27]. The formation of the *4n*AT population was not only important for studying biological evolution, but was also the important step in producing sterile triploid fish by inter-ploid hybridization [10]. The sterile triploid fish exhibited strong disease resistance, fast growth rate, and reduced environmental risk for its sterility, which acted as a perfect aquatic species being widely cultured in China, and obtained great economic benefits [10]. However, the premise of the above described advantages is clarifying the reproductive characteristics of *4n*AT at the morphological and molecular levels.

In this study, we compared the ovarian miRNA profiles of RCC and *4n*AT, and found that the expressions of different miRNAs were varied from 5 to 150,000 reads. The highly expressed miRNAs, i.e., >70,000 reads on average, were closely correlated with ovary maturation. For example, miR-202-5p, which is abundant in both groups of samples, has been reported as a gonad-specific miRNA in frogs [28], Atlantic halibut [29], humans, mice [27], rats [30, 31], and rainbow trout [25]. Analysis of miRNA profiles in the complete ovary development and maturation process in rainbow trout revealed that miR-202-5p expression is much higher in the stage prior to germinal vesicle breakdown compared to any other stage during the process [25]. Other miRNAs, including miR-143a-3p, miR-miR-126-3p, and miR-101-3p, are also abundant in the final oocyte maturation in rainbow trout [25, 32]. Moreover, the second most abundant miRNA in this study, miR-22-3p, has a complex interplay with steroids (estrogen and androgen) [33]. Differential expression analysis revealed that the expression of the gonad development-related miRNAs listed above did not differ significantly between the two groups of samples. These results are consistent with the reproductive characteristics of *4n*AT. Both of the fishes are sexually matured at the age of 1 year and have a similar ovary development process [8].

Non-additive gene expression patterns in polyploid plants or animals have been widely studied for response to genomic shock coupled with the balancing of more than two sets differential chromosomes in one nucleus [5, 7]. Global analysis of miRNA transcriptomes in *Squalius alburnoides*, comparing diploid and triploid hybrids, indicated that the diploid and triploid hybrids share most of their miRNA expression profiles, and the triploid hybrids tend to regulate gene expression to a diploid state [34]. Comparative analysis of miRNAs profiles in this research revealed that majority of miRNAs, obtained from sequencing, displayed no significant differential expression between diploid RCC and *4n*AT. These results might be correlated with the non-additive gene expression patterns in polyploids. Among the differentially expressed miRNAs, the majority were up-regulated. For the miRNA is transcribed based on the DNA templates, this phenomenon might be caused by the double size of the DNA alleles in the allotetraploid hybrid. DNA methylation and miRNA are both important negative epigenetic regulators of gene expression. Xiao et al. [35] have reported that, compared with the parents, the genomic DNA in allotetraploid hybrids displayed hypermethylation levels. Studies in other polyploid species, such as *Brassica* [36], wheat [37], and *Arabidopsis* [38] demonstrated a similar situation in genomic DNA methylation. For instance, the differentially expressed miRNAs might be correlated with gene expression, which coupled with the change of phenotype, in the different ploidy animals.

Cacalier-Smith et al. found that genome size is generally correlated with cell volume and nuclear volume [39]. The surface area of the nuclear envelope available for nucleocytoplasmic transport of RNA is crucial for determining the cell growth rate and metabolism [39]. During evolution, the balance between DNA content and cell volume has been adjusted to allow reasonable growth rate. The cell geometry in polyploidies, large cells tending to have smaller surface area to volume ratios, would reduce the available nuclear envelope for nucleocytoplasmic transport, thus limiting the metabolism and growth rate. However, these effects of polyploidization at the phenotypic level are not exact and universal, which also depend on the environment [40]. The allotetraploid hybrids used in this study displayed a smaller body size and slower growth rate, although the volume of blood cell nuclei and the size of gametes were larger than in the diploid parental fish [8, 10]. Annotation of genes targeted by differentially expressed miRNAs with public databases indicated that the function of target genes mainly focused on macromolecular metabolism and transport, cellular skeleton, defense mechanisms, and transcription. Variations in morphological traits are governed by complex and well-balanced programs of gene activation and silencing [41]. The functions of target genes of differentially expression miRNAs were consistent with the morphological trait variations in the allotetraploid hybrids and may have a molecular basis for these stable phenotypical changes caused by polyploidy. Moreover, global DNA methylation analysis in *4n*AT also revealed that sequences involved in metabolism and disease resistance displayed DNA methylation variation, which is consistent with the miRNA profile results and phenotypic change [35].

## Conclusions

This is the first study to describe the expression profiles and involvement of miRNAs in gene regulation coupled with polyploidy in ovary tissues. Molecular evidence revealed that the ovarian development process is similar in diploid RCC and 4nAT, whereas the differential expressions of miRNAs and mRNAs are mainly caused by ploidy change. Therefore, our results provide strong epigenetic evidences for the fertility of female allotetraploid fish and phenotypical changes caused by polyploidy.

## Methods

### Ethics statement

All experiments were approved by Animal Care Committee of Hunan Normal University and carried out according to the Care and Use of Agricultural Animals in Agricultural Research and Teaching Guidelines, approved by the Science and Technology Bureau of China. The fish were deeply anesthetized with 100 mg/L MS-222 (Sigma, St. Louis, MO, USA) prior to dissection.

### Animals and sample collecting

RCC and *4n*AT used in this experiment were cultured in ponds at the Protection Station of Polyploidy Fish, Hunan Normal University, and fed with artificial feed once per day. One-year-old fish were sampled in April. The ploidy of fish was determined by flow cytometry analysis as previously described. The fish were then anesthetized and dissected. The ovaries were removed from the fish, frozen in liquid nitrogen, and stored at −80 °C prior to RNA isolation. Three fish of each type were collected and analyzed.

### Total RNA isolation

Total RNA isolation was performed with TRIzol® reagent (Invitrogen, Carlsbad, CA, USA) according to the manufacturer’s instructions. In brief, 100 mg of ovary tissue was homogenized with 1 ml TRIzol with a glass homogenizer, and then centrifuged at 12,000 × g for 10 min at 4 °C to discard the precipitate. Purification with phenol/chloroform (BBI, Shanghai, China) was then carried out followed by precipitation with isopropyl alcohol (BBI, Shanghai, China) and dissolution in RNase-free water. The quality and concentration of total RNA were verified by 1.5 % agarose electrophoresis and detecting the ratio of 260/280 by spectrophotometry (Eppendorf, Westbury, NY, USA). The integrity of total RNA was evaluated with an Agilent 2100 Bioanalyzer (Agilent Technologies, Palo Alto, Calif., Germany).

### MiRNA library construction and sequencing

A small RNA library was constructed with a TruSeq Small RNA Sample Preparation Kit (Illumina, San Diego, CA, USA) according to the manufacturer’s instructions based on the special structural characteristics of small RNAs, with a phosphate group in the 5’ left and a hydroxyl in the 3’ left. Using total RNA as the initial sample, the small RNA was ligated to a 3’ adapter at first, then a 5’ adapter following the addition of the reverse transcription primer to create cDNA constructs. After PCR amplification with forward and reverse primers complementary to the 5’ and 3’ adapters, the products were purified with 6 % PAGE and selected with the proper size to construct the small RNA library. The effective concentration of the small RNA library was verified by Qubit 2.0 (Invitrogen, Carlsbad, CA, USA) and Q-PCR. The insert size of the library was evaluated with an Agilent 2100 Bioanalyzer (Agilent Technologies, Palo Alto, Calif., Germany). The qualified small RNA libraries were sequenced with the HiSeq 2500 SE50 system (Illumina, San Diego, CA, USA).

### Bioinformatic analysis

Clean reads were obtained by filtering low quality reads and selecting 18–30 nt reads from the raw reads for the further analysis. The clean reads were searched against the GenBank, Rfam, and ZFIN databases to discard the annotated non-coding RNA reads and find the reads mapped to the reference genome for miRNA identification. The remaining sequences were subsequently used to identify the conserved and novel miRNAs with miRDeep2 software [42]. To analyze the differential expression of miRNAs in RCC and *4n*AT ovaries, miRNA reads were then normalized to obtain the miRNA expression using the DEGseq software (http://www.bioconductor.org/packages/release/bioc/html/DEGseq.html). To understand the function of differentially expressed miRNAs, potential target genes for miRNAs were predicted by the miRanda database, then annotated and the functional modules identified by BLAST searches against the NR, Swiss-Prot, GO, COG, and KEGG databases. The correlation analysis of biological replicates was assessed with the Pearson correlation coefficient (R^2^) with pairwise comparisons >0.92.

### Quantitative real-time PCR

To validate the differential expression of miRNA identified from the sequencing results, the relative expressions of miRNAs were evaluated by quantifying the miRNA stem-loop. The total RNA was used to obtain cDNA with a reverse transcription kit (Invitrogen, Carlsbad, CA, USA) with specific primers for each miRNA. The PCR reaction was performed using ABI SYBRGreen PCR master mix (Applied Biosystems, Foster City, CA, USA) on the ABI 7500 PCR system (Applied Biosystems, Foster City, CA, USA) with specific primers (Table 1). β-actin was used as the internal control. For each sample, three independent repetitions were tested.

**Table 1:** Specific primers used in Q-PCR

Primer	Sequence
miR-6843-3p-RT	CTCAACTGGTGTCGTGGAGTCGGCAATTCAGTTGAGCAGCAT
miR-6843-3p-F	ACACTCCAGCTGGGTTGGTCTCTGTA
miR-81b-5p-RT	CTCAACTGGTGTCGTGGAGTCGGCAATTCAGTTGAGACTGTGA
miR-81b-5p-F	ACACTCCAGCTGGGCCGGGTGTGTGTT
miR-42-5p-RT	CTCAACTGGTGTCGTGGAGTCGGCAATTCAGTTGAGACTGTGA
miR-42-5p-F	ACACTCCAGCTGGGCTGGGTGTGTGCT
miR-21-5p-RT	CTCAACTGGTGTCGTGGAGTCGGCAATTCAGTTGAGGCCAACAC
miR-21-5p-F	ACACTCCAGCTGGGTAGCTTATCAGACT
miR-2368-3p-RT	CTCAACTGGTGTCGTGGAGTCGGCAATTCAGTTGAGAAAAAGCC
miR-2368-3p-F	ACACTCCAGCTGGGGCTGTCAGAAAGGG
Actin-RTF	TCTACAACGAGCTGCGTGTTG
Actin-RTR	CCTGTTGGCTTTGGGATTGA

### Statistical analysis

The quantitative real-time PCR data were expressed as means ± SD and the significant differences were confirmed by t-tests in SPSS 13.0 software. *P*-values < 0.05 and < 0.01 were taken to indicate a statistical difference.

### Availability of supporting data

All the short read sequences were available in the NCBI Sequence Read Archive (http://www.ncbi.nlm.nih.gov/sra) with a study number SRX1233598 and SRX1239635.

## Additional files

Additional file 1: Table S1. Data statistics of samples. **Table S2.** Classify annotation of clean reads. **Table S3.** Actual reads counts of miRNAs in different samples derived from sequencing. **Table S4.** Statistic of correlation by pairing comparision. **Table S5.** Differential expressed miRNAs and change fold. **Table S6.** Function annotation and classification of genes targeted by differential expressed miRNAs. **Table S7.** Function classfication of targeted genes of differential expressed miRNAs with KEGG pathway database. (XLS 248 kb) Additional file 2: Figure S1. Correlation analysis of biological repeats between the RCC and *4n*AT groups. (DOC 81 kb)

## Abbreviations

RCC: Red crucian carp
CC: Common carp
*4n*AT: The allotetraploid hybrids
