# Supplementary material for: MicroRNA profiles reveal female allotetraploid hybrid fertility
Source: BMC Genet. 2015 Oct 14;16:119. doi: 10.1186/s12863-015-0276-y (PMC4607245; doi:10.1186/s12863-015-0276-y)
Supplement: Additional file 2: Figure S1. — Correlation analysis of biological repeats between the RCC and 4nAT groups. (DOC 81 kb) [file 12863_2015_276_MOESM2_ESM.doc]

**
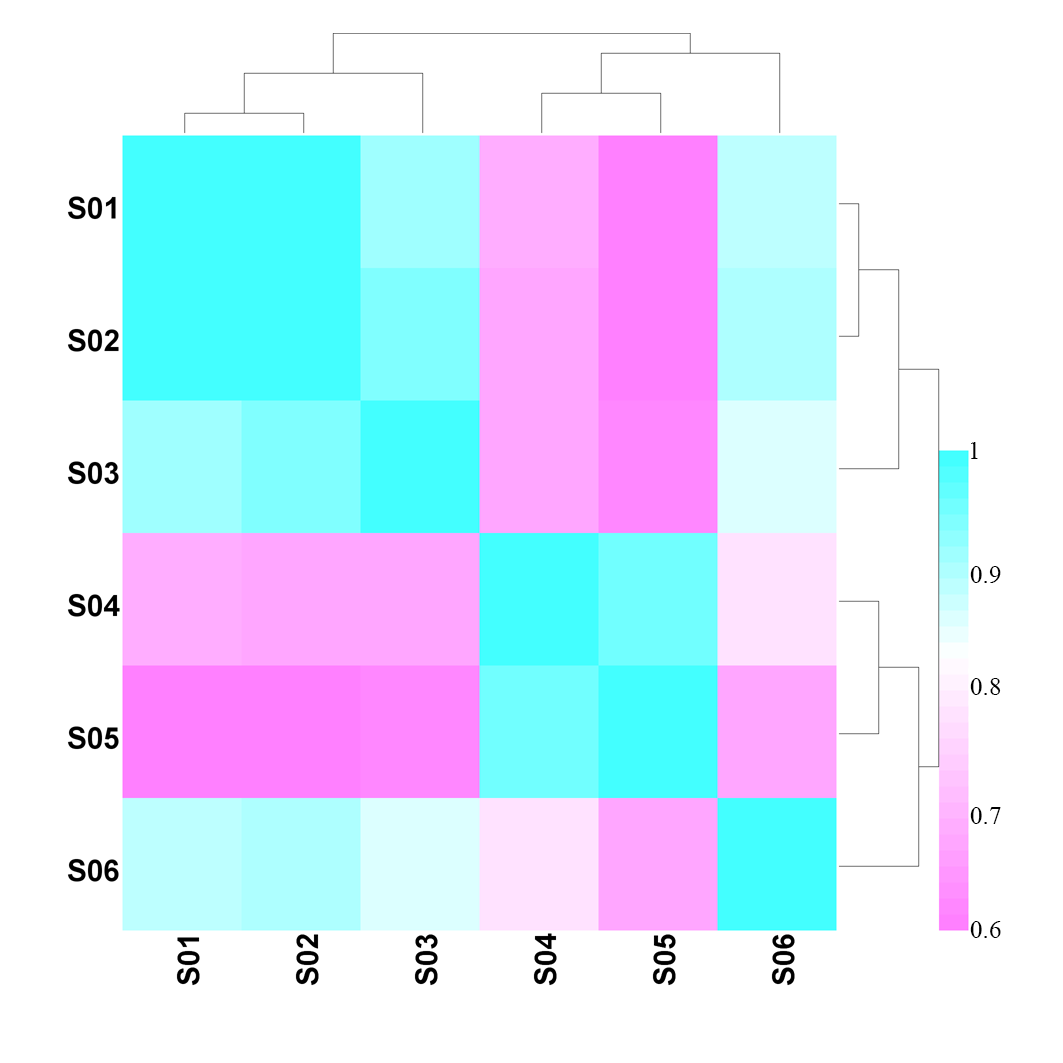
**

Figure S1 Correlation analysis of biological repeats between the RCC and *4n*AT groups. The diploid RCC group contains S01, S02 and S03. The allotetraploid hybrids (*4n*AT) group contains S04, S05 and S06. The color gradually changed from pink to blue represents correlation coefficient varied from 0.6 to 1.0.
